# Vibration response imaging: protocol for a systematic review

**DOI:** 10.1186/2046-4053-2-86

**Published:** 2013-09-25

**Authors:** Marc P Berry, Luigi Camporota, George Ntoumenopoulos

**Affiliations:** 1 National Institute for Health Research (NIHR) Biomedical Research Centre, Guy’s and St Thomas’ NHS Foundation Trust and King’s College London, 16th Floor Tower Wing, Guy’s Hospital, London SE1 9RT, UK; 2 Division of Asthma, Allergy and Lung Biology, King’s College London and Department of Adult Critical Care, Guy’s and St Thomas’ NHS Foundation Trust, 5th Floor Tower Wing, Guy’s Hospital, London SE1 9RT, UK; 3 Australian Catholic University, PO Box 968, North Sydney, NSW 2059, Australia

**Keywords:** Vibration response imaging, Lung sound distribution, Lung sound monitoring

## Abstract

**Background:**

The concept of lung sounds conveying information regarding lung physiology has been used extensively in clinical practice, particularly with physical auscultation using a stethoscope. Advances in computer technology have facilitated the construction of dynamic visual images derived from recorded lung sounds. Arguably, the most significant progress in this field was the development of the commercially available vibration response imaging (VRI) (Deep Breeze Ltd, Or-Akiva, Israel). This device provides a non-invasive, dynamic image of both lungs constructed from sounds detected from the lungs using surface sensors. In the literature, VRI has been utilized in a multitude of clinical and research settings. This systematic review aims to address three study questions relating to whether VRI can be used as an evaluative device, whether the images generated can be characterized, and which tools and measures have been used to assess these images.

**Methods/Design:**

This systematic review will involve implementing search strategies in five online journal databases in order to extract articles relating to the application of VRI. Appropriate articles will be identified against a set of pre-determined eligibility criteria and assessed for methodological quality using a standardized scale. Included articles will have data extracted by the reviewers using a standardized evidence table. A narrative synthesis based on a standardized framework will be conducted, clustering evidence into three main groups; one for each of the study questions. A meta-analysis will be conducted if two or more research articles meet pre-determined criteria that allow quantitative synthesis to take place.

**Discussion:**

This systematic review aims to provide a complete overview of the scope of VRI in the clinical and research settings, as well as to discuss methods to interpret the data obtained from VRI. The systematic review intends to help clinicians to make informed decisions on the clinical applicability of the device, to allow researchers to identify further potential avenues of investigation, and to provide methods for the evaluation and interpretation of dynamic and static images. The publication and registration of this review with PROSPERO provides transparency and accountability, and facilitates the appraisal of the proposed systematic review against the original design.

**Trial registration:**

PROSPERO registration number: CRD42013003751

## Background

The concept that sounds generated by the lungs during the act of breathing have distinguishing features is not a new concept [1,2]. The skill of auscultation is based on recognizing these distinguishing sounds and translating this information into clinical meaning [3]. Notoriously, auscultation has limitations due to its subjectivity, ambiguity and potential for error [3,4]. With advancements in computer-based technology, it was realized that these respiratory sounds could be recorded, and in turn, processed to form visual representations that could be quantified [5,6]. It was theorized that if respiratory sounds could be visually represented then clinical meaning could be derived from these images [5,6]. Furthermore, unlike auscultation, images could compare sounds in both lungs simultaneously [7]. Many endeavors have been made into how best to image the lungs in this way [5,6]. However, arguably the largest progression in this field has come about with the commercialization of the imaging technology, vibration response imaging (VRI) (Deep Breeze Ltd, Or-Akiva, Israel) [8].

VRI is a non-invasive, radiation-free imaging device, which utilizes vibration energy of sound created by the lungs during breathing [9]. The device creates a dynamic image, which is representative of the distribution of this sound vibration energy, via an array of active sensors [9,10]. Each of these sensors simultaneously record a short 12 to 20 second sound waveform clip (frequencies of 50 to 400 Hz) from the area of the posterior chest wall it overlays [11]. These recordings are converted to digital signals, filtered between 150 and 200 Hz (to minimize artefact such as heart sounds) and finally represented by a series of greyscale images; each representing 0.17 seconds of recording [9,12]. Due to its ability to visually display lung sounds and provide quantitative data, VRI has sparked a great deal of interest in a wide range of clinical and research settings, relating to respiratory care and respiratory medicine [9,13-17].

VRI has clinical appeal as an imaging technique as it can be applied relatively easily by an array of health professionals involved in respiratory care, including doctors and physiotherapists [16]. It allows practitioners to make decisions based on near real-time images, online and offline, using technology that provides no risk to the patient; which are the main disadvantages of computed tomography (CT) and chest radiography [8,9].

To date, research into the application and evaluation of VRI as an imaging tool has been varied within respiratory care. It has been demonstrated that lung sounds can be visually characterized in healthy individuals [11,18], and that the signature of lung sounds may change according to pathology or disease state, which again may be categorized [10,13,19,20]. Furthermore, the ability of VRI to evaluate physiological changes and infer clinical response to an intervention has been explored, both in self-ventilating [10,21] and mechanically ventilated patients [16,22]. Naturally, comparisons have been made against ‘gold standard’ imaging technologies (that is, CT) in order to establish levels of sensitivity and specificity [10,15]. Reproducibility of images, intra-rater reliability and inter-rater agreement when interpreting images have also been routes of exploration [11,20]. The body of evidence exploring VRI has been considerably varied in respect to the methodology, its application, the population of interest, the visual characterization of clinical signs, and the interpretation of the dynamic and static images. These endeavors have been in order to establish VRI as a clinically relevant imaging device that has the advantage of providing non-invasive, near real-time and dynamic images at the patients’ bedside [23].

The aim of this systematic review is to synthesize results from the body of literature surrounding VRI and its use, and to generate recommendations regarding three study questions:

1. Can lung sounds in healthy individuals, as well as those in individuals with lung pathology/disease be characterized using VRI in the adult population?

2. Can VRI be used to evaluate changes in lung sound distribution in response to an intervention or to evaluate lung sound distribution changes over time in the adult population?

3. What tools or measures have been used to interpret VRI lung sound data in the adult population?

## Methods/design

### Selection criteria and eligibility

#### Study and publication characteristics

*A priori* information derived from the VRI literature suggests a lack of randomized controlled trials (RCTs) in this area. Due to this, all other interventional and observational research designs and methodologies will be considered in this systematic review. Study design and publication types to be excluded are: Cochrane reviews, systematic reviews, opinion articles, editorials and narrative articles. Book chapters, device manuals and guidance notes will also be excluded from this review.

#### Participant characteristics

Participants to be included are human adult participants over 16 years old. Restrictions will not be placed on types of lung condition, disease state (including healthy individuals), type of ventilation, clinical intervention or procedure. Furthermore, restrictions will not be placed on gender or ethnic background of participants.

#### Intervention characteristics

Studies using the VRIxv and VRIxp devices developed by Deep Breeze will be reviewed. No restrictions will be placed on how the VRI device is used for respiratory sound imaging. However, studies using VRI to detect non-respiratory sounds (for example, cardiac sounds) will not be considered eligible for inclusion. Furthermore, studies using sound imaging devices other than VRI will not be included in this review.

### Search strategy

Search terms and keywords were established *a priori*, and used to construct database search strategies (Appendix A) and inform manual searches. Search strategies will be conducted in MEDLINE (Ovid), Embase (Ovid), CINAHL (EBSCOhost), CENTRAL and Web of Science. Publication date will be restricted to articles published after 2005 (first year that research using VRI began). Publication status restrictions or language restrictions will not be applied at this point.

Manual searches for further potential articles will be conducted through a process of reviewing the reference lists of eligible articles, and using the search terms and keywords from the search strategies.

Sources of grey literature (for example, unpublished articles, articles in press), sources known to experts in the field and abstracts from major respiratory conferences (International Symposium on Intensive Care and Emergency Medicine (ISICEM), European Society of Intensive Care Medicine (ESICM), American Thoracic Society (ATS)) since 2005 will also be reviewed for potentially eligible articles.

Each search strategy carried out will be systematically and electronically tabulated, documenting in which database the search strategy was conducted, the date that the search was carried out and the number of ‘hits’ that each search strategy returned, including references for each ‘hit’. Each database and data source will have a worksheet dedicated to it to avoid confusion. Manual searches will also be electronically tabulated; documenting titles of the source articles or resource searched, and search dates. Searches of websites or search engines will be documented, providing the website (and URL), the search terms used and the date on which the search was carried out. Documentation of references sourced from relevant research articles and published resources (for example, equipment handbooks, book chapters) will include the source article/resource reference, date of the reference list search and references of articles sourced. All publications will then be managed and stored via the electronic referencing programme, EndNote X6 (Thomson Reuters, New York, NY, USA) and EndNote Web Sync through designated groups, with any duplicate articles removed.

### Study selection

**Figure 1:**
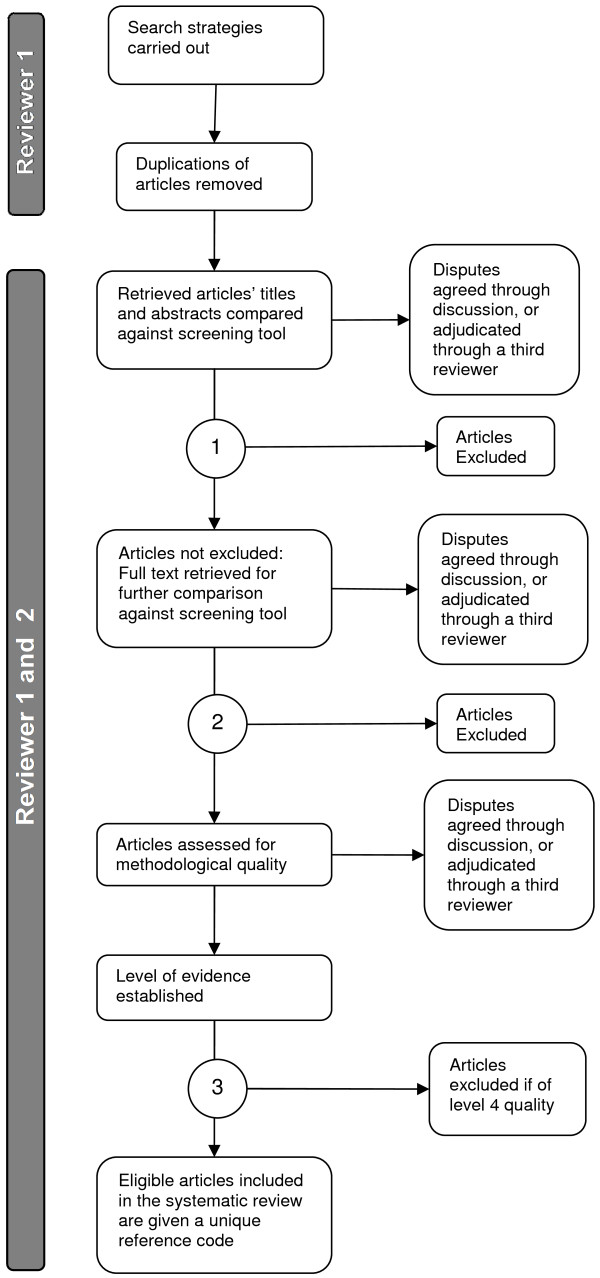
**Flowchart describing the review process of articles sourced by the search strategy.** The review process will be carried out by reviewer 1 (MB) and reviewer 2 (GN). The circled numbers 1, 2 and 3 depict the points at which articles will be tagged with codes in EndNote X6 to determine their group allocation.

All the articles sourced will be placed in a designated group, which will be duplicated for each reviewer to assess from. The two reviewers (MB and GN) will independently review each duplicate group of articles against the eligibility criteria (using a standardized screening tool) in order to derive eligible articles, initially based on the title and abstract (Figure 1). The reviewers will then compare their decisions regarding the eligibility of articles. Once consensus has been reached, articles will be tagged in EndNote with a pre-determined code which will allocate articles to one of two groups: potentially eligible/not definitively excluded articles or excluded articles. If consensus cannot be reached, a third reviewer (LC) will adjudicate to which group articles will be allocated (circled number 1, Figure 1). If non-English articles have an English title and/or abstract, this will be assessed using the screening tool to deem eligibility for inclusion. If the study is suitable, only the abstract will be considered for inclusion. Due to limitations of resources, the full text will not be translated. The reviewers will then assess the full text of the articles in the potentially eligible/not definitively excluded group against the standardized screening tool in the same manner as previously described (circled number 2, Figure 1), allowing a group of eligible articles to be identified for methodological quality review.

### Data extraction

The final included articles in the systematic review will be grouped together and assigned a unique reference code. Data extraction will be performed for each eligible article by the two reviewers (MB and GN) independently, tabulating data in a standardized evidence table. Disputes will again be resolved through discussion between reviewers, with third reviewer (LC) adjudication if necessary.

Data relating to the following areas will be extracted to the evidence table (based on information from the Centre for Reviews and Dissemination (CRD) [24]:

•Consensus decided level of evidence;

•Study characteristics: study aims and objectives, study design, eligibility criteria, recruitment procedures used, country, year, and sample size;

•Participant characteristics: age, gender, disease characteristics, and the number of participants in each study group which were eligible, enrolled, studied, withdrawn and/or lost to follow-up;

•Intervention and setting: study and intervention delivery setting, intervention and control descriptions, and any theoretical basis information for interventions;

•Analysis tools: type of tool or measurement used for VRI data analysis;

•Outcome data: outcome measures used, statistical methods used, and appropriateness of these methods and summary outcome data;

•Results: analysis type and results of study analysis;

•Main conclusions: key conclusion points and appropriateness of conclusion;

•Other key information: funding source, conflicts of interest, costs, resources used and adverse events*.*

Information collected through the critical appraisal checklist can be transcribed to the evidence table. Data not collected through the methodological quality review process will be collected separately, if necessary. Correspondence with authors will be established to clarify or expand on data published in an article if it is unclear or missing. The third reviewer will adjudicate any disagreements, allowing consensus to be reached.

Key outcomes of interest have been identified *a priori* to enable data synthesis and potential meta-analysis (if appropriate) for study questions 1 and 2. The key outcomes of interest are as follows:

•Vibration energy distribution (%);

•Vibration energy amplitude (AU);

•Geographical distribution and intensity of standard 256 greyscale coded pixels of VRI images.

Descriptions of VRI waveform patterns, and of static and dynamic VRI image presentations will be considered for analysis, where available and relevant to the study questions.

### Methodological quality review

Methodological quality of eligible articles will be assessed by each reviewer (MB and GN) independently using the Scottish Intercollegiate Guidelines Network (SIGN) critical appraisal checklists and notes, in duplication [25]. Each study will be assessed against a critical appraisal checklist specific to the study design or a data extraction checklist for non-represented study designs. Any disputes will be resolved through discussion between reviewers, with adjudication of a third reviewer (LC) if necessary. This decision making process will also be documented in a pre-determined evidence table. Once consensus has been reached, a level of evidence will be decided upon, based on the grading system described by SIGN [26]. Articles of level 4 evidence will be excluded from the systematic review at this point (circled number 3, Figure 1).

### Data analysis and synthesis

A meta-analysis will be conducted by the reviewers (MB and GN) if two or more articles [27] of evidence level 1++, 1+, 2++ or 2+ [26], which are deemed to be similar enough in study design, patient population and outcome measures are identified [27]; then a quantitative synthesis will be conducted. A random effects model of meta-analysis will be used. Cochran’s Q and I^2^ will be calculated to assess heterogeneity of data [27]. Statistical parameters relating to the key outcomes of interest have been identified for study questions 1 and 2. For study question 1, parameters include levels of agreement and correlations between VRI and other lung imaging devices or lung function measurements. Sensitivity, specificity, positive predictive value, negative predictive value and absolute error will be considered for study question 1 when evaluating the accuracy of characterization of VRI images/data. Inter-rater reliability and inter-rater agreement shall also be explored for study question 1, in relation to the accuracy of VRI image characterization and interpretation.

For study question 2, intraclass correlations and mean differences relating to the key outcomes will be analyzed where appropriate. The results of statistical testing for repeated measures (for example, analysis of variance (ANOVA), Wilcoxon signed-rank test) and paired data (for example, *t*-test) will also be compared for study question 2 if outcome data is homogeneous in nature. These may be used either to show data stability between VRI images or a change in data, either over time or in response to an intervention. Descriptive statistics relating to the key outcomes of interest for study questions 1 and 2 will include mean (± SD) or median (IQR). For each statistical parameter, *P* values and 95% confidence intervals will be analyzed and pooled, where applicable and appropriate.

Pooling of the appropriate key outcomes of interest and their associated statistical parameters will be carried out and presented in a forest plot separately for study question 1 and 2. Statistical analysis of pooled outcomes will follow the statistical testing method used by the authors in the articles from which the outcome measurements are derived. This will be synthesized using the open source programme OpenMeta[Analyst] (Center for Evidence-Based Medicine, Brown University, Providence, RI, USA). Any statistical analysis that is deemed appropriate will be carried out using SPSS (IBM SPSS Statistics 20, Armonk, NY, USA). *A priori* information suggests that the articles surrounding VRI are small trials and may lack the measurement of an effect size [28]. Therefore the use of funnel plots in assessing reporting bias may not be appropriate.

If quantitative pooling of studies is not possible, the evidence will be synthesized narratively according to the standardized synthesis framework described by the CRD [24].

#### Developing a theory

Theories relating to VRI will be based on the study questions. A cluster group of data will be formed and assigned to each of the three study questions (VRI image characterization, VRI used as an evaluative device, and tools and measures used to assess VRI images). The data in each cluster group will be synthesized separately, in order to focus the answering of the study questions. Data in these cluster groups will be sourced from the standardized evidence table.

#### Developing a preliminary synthesis

Extracted data will be allocated to a main cluster group and tabulated, allowing transparency of article comparison. Data from articles may appear in several or all cluster groups as different aspects of the extracted data being synthesized.

#### Exploring relationships within and between studies

Assigning articles to three main pre-defined clusters will facilitate the comparison of study characteristics and identification of similar methodologies between studies. Furthermore, the outcomes of interest and their associated statistical parameters will be used to make comparisons between articles collated in each cluster. Further subgroups within these clusters can be subsequently identified through ‘idea webbing/conceptual mapping’ [24] carried out using Mindjet Version 11.2.185 (Mindjet, San Francisco, CA, USA), providing clear documentation of the development of subgroups. Subgroup data will be tabulated to allow for comparisons to be made between similar outcomes of interests and/or their associated statistical parameters, and to assist in clarifying whether a meta-analysis is feasible. Graphical representation of data may be possible (for example, forest plots, L’Abbé plots) if data and methodologies are sufficiently homogeneous. It will be at this point that a decision regarding the synthesis of data via meta-analysis will be made.

#### Assessing the robustness of the synthesis, conclusions and recommendations

At this stage of synthesis, a consensus decision on the robustness of the synthesis in each of the three clusters will be made between the reviewers (MB and GN). This process of critical reflection will be facilitated and documented via an adapted version of SIGN’s considered judgment on quality of evidence form and guidance notes [29]. From this, a summary for key aspects of the body of evidence in each cluster, as well as any other factors that may have been considered during the review process will be documented along with a grade of recommendation, as stated by SIGN [26].

## Discussion

The aim of this systematic review is to provide an overview of the scope of VRI in both the clinical and research settings. It will address the extent to which VRI has been used to diagnose specific lung pathologies, to evaluate change in lung characteristics, both in the short- and long-term, and to what extent lung sound images can be characterized in relation to lung state or pathology. Furthermore, it aims to highlight the different methods used to interpret VRI data in the literature. The systematic review intends to help clinicians to make informed decisions on the clinical applicability of the device, to allow researchers to identify further potential avenues of investigation, and to provide methods for the evaluation and interpretation of dynamic and static images. The publishing of this protocol, along with its registration with PROSPERO [30] provides transparency and accountability for the systematic review. It will also serve as a reference document to readers of the final systematic review publication, facilitating critical appraisal of its conduct in relation to the original protocolized design.

The findings of the systematic review will be disseminated through publication in a peer reviewed journal, relevant to the subject matter. Reporting of the systematic review will follow recommendations described in the Preferred Reporting Items for Systematic Reviews and Meta-Analyses (PRISMA) statement [31], and will be formatted as per specific journal publication guidelines.

## Appendix A

Database search strategies

## MEDLINE and Embase search strategy

The same search strategy was carried out in MEDLINE (Ovid) and Embase (Ovid):

1) vibration* response imag*.mp.

2) limit 1 to yr=“2005–Current”.

3) vri.mp.

4) limit 3 to yr=“2005-Current”.

5) vibration* response device*.mp.

6) limit 5 to yr=“2005-Current”.

7) computer* lung sound*.mp.

8) limit 7 to yr=“2005-Current”.

9) acoustic lung.mp.

10) limit 9 to yr=“2005-Current”.

11) acoustic based lung.mp.

12) limit 11 to yr=“2005-Current”.

13) acoustic based imag*.mp.

14) limit 13 to yr=“2005-Current”.

15) thoracic sound*.mp.

16) limit 15 to yr=“2005-Current”.

17) lung sound distribution.mp.

18) limit 17 to yr=“2005-Current”.

19) computer* lung.mp.

20) limit 19 to yr=“2005-Current”.

21) visual* lung sound*.mp.

22) limit 21 to yr=“2005-Current”.

23) breath sound* distribution*.mp.

24) limit 23 to yr=“2005-Current”.

25) gr?y scale cod*.mp.

26) limit 25 to yr=“2005-Current”.

27) 2 or 4 or 6 or 8 or 10 or 12 or 14 or 16 or 18 or 20 or 22 or 24 or 26.

For the aforementioned search strategy, the following applies: MEDLINE: (mp=title, abstract, original title, name of substance word, subject heading word, keyword heading word, protocol supplementary concept, rare disease supplementary concept, unique identifier); Embase: (mp=title, abstract, subject headings, heading word, drug trade name, original title, device manufacturer, drug manufacturer, device trade name, keyword).

## CINAHL search strategy

S1) “vibration* response imag*”.

S2) “VRI”.

S3) “vibration* response imag*”.

S4) “computer* lung sound*”.

S5) “acoustic lung”.

S6) “acoustic based lung”.

S7) “acoustic based imag*”.

S8) “thoracic sound*”.

S9) “lung sound distribution”.

S10) “computer* lung”.

S11) “visual* lung sound*”.

S12) “breath sound* distribution*”.

S13) “gr?y scale cod*”.

S14) S1 or S2 or S3 or S4 or S5 or S6 or S7 or S8 or S9 or S10 or S11 or S12 or S13.

Search options: limiters, published date from 1 January 2005 to 31 December 2013; expanders, also search within the full text of the articles; search modes, Boolean/phrase.

## CENTRAL search strategy

#1) “vibration* response imag*” from 2005 to 2013 (word variations have been searched).

#2) “VRI” from 2005 to 2013 (word variations have been searched).

#3) “computer* lung sound*” from 2005 to 2013 (word variations have been searched).

#4) “acoustic lung” from 2005 to 2013 (word variations have been searched).

#5) “acoustic based lung” from 2005 to 2013 (word variations have been searched).

#6) “acoustic based imag*” from 2005 to 2013 (word variations have been searched).

#7) “thoracic sound*” from 2005 to 2013 (word variations have been searched).

#8) “lung sound distribution” from 2005 to 2013 (word variations have been searched).

#9) “computer* lung” from 2005 to 2013 (word variations have been searched).

#10) “visual* lung sound*” from 2005 to 2013 (word variations have been searched).

#11) “breath sound* distribution*”.

#12) “gr?y scale cod*” from 2005 to 2013 (word variations have been searched).

#13) #1 or #2 or #3 or #4 or #5 or #6 or #7 or #8 or #9 or #10 or #11 or #12 from 2005 to 2013 (word variations have been searched).

## Web of Science search strategy

Title=(“vibration* response imag*” or “vri” or “vibration* response device*” or “vibration* response imag*” or “computer* lung sound*” or “acoustic lung” or “acoustic based lung” or “acoustic based imag*” or “thoracic sound*” or “lung sound distribution” or “computer* lung” or “visual lung sound*” or “breath sound* distribution*” or “gr?y scale cod*”).

Timespan, 2005 to 2013; search language, English.

## Abbreviations

ANOVA: Analysis of variance; ATS: American Thoracic Society; CRD: Centre for Reviews and Dissemination; CT: Computed tomography; ESICM: European Society of Intensive Care Medicine; IQR: Interquartile range; ISICEM: International Symposium on Intensive Care and Emergency Medicine; PRISMA: Preferred Reporting Items for Systematic Reviews and Meta-Analyses; RCTs: Randomized controlled trials; SD: Standard deviation; SIGN: Scottish Intercollegiate Guidelines Network; VRI: Vibration response imaging.

## Competing interests

MB and LC declare that they have no competing interests. GN has previously received support from Deep Breeze with the loan of a VRIxv to carry out a published pilot study [16].

## Authors’ contributions

MB, GN and LC developed the design of the study. MB developed the search strategies in conjunction with the library service at King’s College London (London, UK). MB wrote the initial draft of the protocol. MB, GN and LC all contributed to revision of the protocol and the current manuscript. The final manuscript was read and approved by MB, GN and LC.

## References

[B1] Pasterkamp H Kraman SS Wodicka GR Respiratory sounds. Advances beyond the stethoscope Am J Respir Crit Care Med 1997 156 974 987 10.1164/ajrccm.156.3.9701115 9310022

[B2] Pasterkamp H Carson C Daien D Oh Y Digital respirosonography. New images of lung sounds Chest 1989 96 1405 1412 10.1378/chest.96.6.1405 2684558

[B3] Mangione S Nieman LZ Pulmonary auscultatory skills during training in internal medicine and family practice Am J Respir Crit Care Med 1999 159 1119 1124 10.1164/ajrccm.159.4.9806083 10194155

[B4] Brooks D Thomas J Interrater reliability of auscultation of breath sounds among physical therapists Phys Ther 1995 75 1082 1088 7501711 10.1093/ptj/75.12.1082

[B5] Kompis M Pasterkamp H Wodicka GR Acoustic imaging of the human chest Chest 2001 120 1309 1321 10.1378/chest.120.4.1309 11591576

[B6] Charleston-Villalobos S Cortes-Rubiano S Gonzalez-Camarena R Chi-Lem G Aljama-Corrales T Respiratory acoustic thoracic imaging (RATHI): assessing deterministic interpolation techniques Med Biol Eng Comput 2004 42 618 626 10.1007/BF02347543 15503962

[B7] Torres-Jimenez A Charleston-Villalobos S Gonzalez-Camarena R Chi-Lem G Aljama-Corrales T Asymmetry in lung sound intensities detected by respiratory acoustic thoracic imaging (RATHI) and clinical pulmonary auscultation Conf Proc IEEE Eng Med Biol Soc 2008 2008 4797 4800 19163789 10.1109/IEMBS.2008.4650286

[B8] Marini JJ Acoustic monitoring–super sonics? Crit Care 2009 13 162 10.1186/cc7908 19664167 PMC2750133

[B9] Dellinger RP Jean S Cinel I Tay C Rajanala S Glickman YA Parrillo JE Regional distribution of acoustic-based lung vibration as a function of mechanical ventilation mode Crit Care 2007 11 R26 10.1186/cc5706 17316449 PMC2151859

[B10] Anantham D Herth FJ Majid A Michaud G Ernst A Vibration response imaging in the detection of pleural effusions: a feasibility study Respiration 2009 77 166 172 10.1159/000168784 18974633

[B11] Maher TM Gat M Allen D Devaraj A Wells AU Geddes DM Reproducibility of dynamically represented acoustic lung images from healthy individuals Thorax 2008 63 542 548 10.1136/thx.2007.086405 18024534 PMC2571960

[B12] Mehta AC Gat M Mann S Madison JM Accuracy of gray-scale coding in lung sound mapping Comput Med Imaging Graph 2010 34 362 369 10.1016/j.compmedimag.2009.12.012 20171843

[B13] Wang Z Jean S Bartter T Lung sound analysis in the diagnosis of obstructive airway disease Respiration 2009 77 134 138 10.1159/000178023 19033680

[B14] Wang Z Xiong YX Computerized lung sound analysis following clinical improvement of pulmonary edema due to congestive heart failure exacerbations Chin Med J (Engl) 2010 123 1127 1132 20529550

[B15] Lev S Glickman YA Kagan I Shapiro M Moreh-Rahav O Dahan D Cohen J Grinev M Singer P Computerized lung acoustic monitoring can help to differentiate between various chest radiographic densities in critically ill patients Respiration 2010 80 509 516 10.1159/000274382 20090286

[B16] Ntoumenopoulos G Glickman Y Computerised lung sound monitoring to assess effectiveness of chest physiotherapy and secretion removal: a feasibility study Physiotherapy 2012 98 250 255 10.1016/j.physio.2011.12.003 22898583

[B17] Radzievsky N Papyan S Kushnir I Gat M Kushnir A Sagie A Agmon Y Estimation of left ventricular function using a novel acoustic-based device Eur J Clin Invest 2012 42 402 410 10.1111/j.1365-2362.2011.02596.x 21950619

[B18] Yosef M Langer R Lev S Glickman YA Effect of airflow rate on vibration response imaging in normal lungs Open Respir Med J 2009 3 116 122 10.2174/1874306400903010116 19834576 PMC2761668

[B19] Dellinger RP Parrillo JE Kushnir A Rossi M Kushnir I Dynamic visualization of lung sounds with a vibration response device: a case series Respiration 2008 75 60 72 10.1159/000103558 17551264

[B20] Bartziokas K Daenas C Preau S Zygoulis P Triantaris A Kerenidi T Makris D Gourgoulianis KI Daniil Z Vibration response imaging: evaluation of rater agreement in healthy subjects and subjects with pneumonia BMC Med Imaging 2010 10 6 10.1186/1471-2342-10-6 20222975 PMC2848624

[B21] Becker HD Slawik M Miyazawa T Gat M Vibration response imaging as a new tool for interventional-bronchoscopy outcome assessment: a prospective pilot study Respiration 2009 77 179 194 10.1159/000182972 19065052

[B22] Lev S Glickman YA Kagan I Dahan D Cohen J Grinev M Shapiro M Singer P Changes in regional distribution of lung sounds as a function of positive end-expiratory pressure Crit Care 2009 13 R66 10.1186/cc7871 19426555 PMC2717423

[B23] Jean S Cinel I Gratz I Tay C Lotano V Deal E Parrillo J Dellinger R Image-based monitoring of one-lung ventilation Eur J Anaesthesiol 2008 25 995 1001 10.1017/S0265021508004481 18492316

[B24] Centre for Reviews and Dissemination (CRD) Systematic Reviews: CRD’s Guidance for Undertaking Reviews in Health Care 2009 York: University of York

[B25] Scottish Intercollegiate Guidelines Network (SIGN) Annex C: Critical Appraisal - Notes and Checklists 2013 Edinburgh: SIGN http://www.sign.ac.uk/guidelines/fulltext/50/annexc.html

[B26] Scottish Intercollegiate Guidelines Network (SIGN) SIGN Grading System 1999 – 2012 2013 Edinburgh: SIGN http://www.sign.ac.uk/guidelines/fulltext/50/annexoldb.html

[B27] Higgins JPT Green S Cochrane Handbook for Systematic Reviews of Interventions 2008 Chichester: Wiley-Blackwell

[B28] Lau J Ioannidis JP Terrin N Schmid CH Olkin I The case of the misleading funnel plot BMJ 2006 333 597 600 10.1136/bmj.333.7568.597 16974018 PMC1570006

[B29] Scottish Intercollegiate Guidelines Network (SIGN) Considered Judgement on Quality of Evidence 2008 Edinburgh: SIGN http://www.biomedcentral.com/content/supplementary/2046-4053-1-17-S4.PDF

[B30] The University of York Centre for Reviews and Dissemination PROSPERO international prospective register of systematic reviews http://www.crd.york.ac.uk/NIHR_PROSPERO/

[B31] Liberati A Altman DG Tetzlaff J Mulrow C Gotzsche PC Ioannidis JP Clarke M Devereaux PJ Kleijnen J Moher D The PRISMA statement for reporting systematic reviews and meta-analyses of studies that evaluate health care interventions: explanation and elaboration PLoS Med 2009 6 e1000100 10.1371/journal.pmed.1000100 19621070 PMC2707010

